# Innate Immune Responses and Viral-Induced Neurologic Disease

**DOI:** 10.3390/jcm8010003

**Published:** 2018-12-20

**Authors:** Yuting Cheng, Dominic D. Skinner, Thomas E. Lane

**Affiliations:** Division of Microbiology & Immunology, Department of Pathology, University of Utah School of Medicine, Salt Lake City, UT 84112, USA; yuting.cheng@path.utah.edu (Y.C.); dominic.skinner@path.utah.edu (D.D.S.)

**Keywords:** virus, innate immunity, neutrophils, demyelination, remyelination

## Abstract

Multiple sclerosis (MS) is a disease of the central nervous system (CNS) characterized by chronic neuroinflammation, axonal damage, and demyelination. Cellular components of the adaptive immune response are viewed as important in initiating formation of demyelinating lesions in MS patients. This notion is supported by preclinical animal models, genome-wide association studies (GWAS), as well as approved disease modifying therapies (DMTs) that suppress clinical relapse and are designed to impede infiltration of activated lymphocytes into the CNS. Nonetheless, emerging evidence demonstrates that the innate immune response e.g., neutrophils can amplify white matter damage through a variety of different mechanisms. Indeed, using a model of coronavirus-induced neurologic disease, we have demonstrated that sustained neutrophil infiltration into the CNS of infected animals correlates with increased demyelination. This brief review highlights recent evidence arguing that targeting the innate immune response may offer new therapeutic avenues for treatment of demyelinating disease including MS.

## 1. Introduction

Multiple sclerosis (MS) is a chronic inflammatory neurodegenerative disease characterized by multifocal regions of central nervous system (CNS) neuroinflammation, demyelination, and axonal loss that ultimately results in extensive neurologic disability [1]. Multifocal demyelinating lesions eventually lead to various clinical symptoms such as impaired motor skills, cognitive decline, behavioral deficits, and vision loss [1,2,3]. Inflammatory T cells reactive to proteins embedded within the myelin sheath are considered important in lesion formation. This notion is supported by preclinical animal models of MS, genome-wide association studies (GWAS) studies and the mechanisms-of-action of FDA-approved disease-modifying therapies that mute clinical relapse by impeding infiltration of activated T cells into the CNS [1,4]. In addition, the success of anti-CD20 therapies in reducing new lesion formation argues for an important role for B cells in contributing to disease [5]. With this in mind, new areas of investigation are focusing on identifying how oligodendrocytes may contribute to disease [6] as well as developing strategies that promote maturation of oligodendrocyte progenitor cells (OPCs) into mature myelin-producing oligodendroglia. Trapp and colleagues [7] have demonstrated OPCs are spread throughout the CNS and appear in high density within some subacute lesions during early stages of MS. Subsequent to OPC maturation, there is limited remyelination leading to the formation of shadow plaques, in which patches of remyelinated white matter are composed of disproportionally thin myelin sheaths surrounding axons [7,8,9,10,11,12,13]. Nonetheless, as disease progresses, there is ultimately remyelination failure that is reflective of an inability of OPCs to mature into myelin-producing oligodendrocytes.

Available evidence indicates that the cause of MS is multifactorial and includes the genetic background of the individual as well as potential environmental influences [4,14,15,16]. Although viruses such as herpes simplex virus type-1 (HSV-1), measles, human T cell leukemia virus type-1 (HTLV-1), human coronaviruses, human herpesvirus-6 (HHV-6), human endogenous retroviruses (HERV), and Epstein Bar Virus (EBV) have been suggested to be associated etiologically with MS, no clear causal relationship between MS and viral infection has been firmly established [17,18,19,20,21,22,23,24,25,26,27]. The development of animal models in which the clinical and histopathology is similar to that observed in the majority of patients is imperative in order to better understand the underlying pathological mechanisms contributing to MS.

## 2. JHMV-Induced Neurologic Disease

Several excellent rodent models of MS have been developed which meet the necessary criteria. The neurotropic JHM strain of mouse hepatitis virus (JHMV) and Theiler’s murine encephalomyelitis virus (TMEV) are two well-accepted models of viral-induced neurologic disease which mimic clinical and histopathological characteristics of MS. TMEV infection of the CNS of susceptible SJL mice results in a persistent infection associated with an immune-mediated chronic demyelinating disease [28]. Herein, we focus on the JHMV model of neurologic disease and cellular contributors to demyelination in persistently infected mice. Intracranial (i.c.) infection of susceptible strains of mice such as C57BL/6 with JHMV results in an acute encephalomyelitis that is accompanied by gray matter involvement with infection of oligodendrocytes, astrocytes, and microglia [29]. With regards to viral infection of the CNS, it is critical for the immune system to rapidly respond to control viral replication and subsequent spread in order to limit neuropathology and long-term damage. Pattern recognition receptors (PPRs) including Toll-like receptors (TLRs) and RIG-I are expressed within the CNS and provide important sentinel functions that aid in initiating innate immune responses following viral infection. Myd88 is an adapter protein that provides a critical role in transmitting signals provided by TLRs that leads to expression of type I IFN (IFN-I) in addition to proinflammatory genes. The importance of Myd88 in host defense following JHMV infection is emphasized in a recent report indicating that infection of Myd88-/- mice increased mortality associated with failure to control viral replication and enhanced neuropathology [30]. Interestingly, CD4+ T cell responses—but not CD8+ T cell responses—were impacted as evidenced by reduced CD4+ T cell recruitment to the CNS and muted IFN-*γ* expression [30].

### 2.1. Secretion of Proinflammatory Cytokines/Chemokines in Response to JHMV Infection of the CNS

In response to JHMV infection of the CNS, there is a rapid synthesis of mRNA transcripts encoding proinflammatory cytokines and chemokines. In situ hybridization has revealed that astrocytes and microglia are responsible for secreting cytokines and chemokines following JHMV infection [31,32], although it is likely that other resident CNS cells e.g., neurons, macrophages, ependymal cells, etc. are also capable of secreting these molecules. The rapid secretion of cytokines and chemokines serves to help control viral replication as well as mobilize and attract cellular components of the innate immune response. Among the cytokines expressed following JHMV infection is IFN-I. In addition to a role in controlling viral replication within the CNS, IFN-I also enhances expression of cytokines and chemokines as well as increasing expression of MHC and costimulatory molecules. Previous studies have highlighted the importance of IFN-I in host defense against neurotropic viruses including West Nile, Sindbis, and vesicular stomatitis virus [33,34,35]. Within the context of CNS infection by JHMV, Bergmann and colleagues [36] clearly demonstrated that type I IFNs are critical in controlling viral replication. Intracranial infection of IFN-I-receptor knock-out mice resulted in increased mortality and impaired ability to control infection that was associated with increased viral replication in glial cells as well as infecting and replicating in defined populations of neurons. Moreover, expression of IFN-I-stimulated genes was impaired, accompanied with reduced expression of MHC class I. Nonetheless, trafficking and accumulation of virus-specific CD8+ T cells was not affected in the absence of IFN-I signaling arguing that IFN-I is not required for T cell survival as has been shown to occur in response to LCMV infection [37]. These findings elegantly demonstrate that IFN-I is responsible for early control of viral replication and tropism that subsequently allows for more effective T cell-mediated protection. More recently, Perlman and colleagues [38] showed that microglia/macrophage activation and production of IFN-I is dependent upon prostaglandin D2 signaling via D-prostanoid receptor 1 (DP1). Additionally, prostaglandin signaling is required for limiting excessive inflammasome activation and increasing survival.

### 2.2. Chemokine Signaling Promotes Immune Cell Infiltration into the CNS

Chemokines are also expressed early in response to JHMV infection of the CNS and have important functional roles in host defense during acute disease. Expression of CXCL1 serves to attract neutrophils to the CNS by signaling through the receptor CXCR2 expressed on the neutrophil cell surface. The importance of attracting neutrophils is highlighted by the demonstration that blocking the migration of these cells via treatment with neutralizing anti-CXCR2 results in increased mortality and impaired ability to control viral replication. Neutrophils contribute to defense through release of matrix metalloprotease 9 (MMP9) which helps increase the permeability of the blood–brain barrier (BBB) ultimately leading to increased infiltration of virus-specific T cells into the CNS [39]. The effect of anti-CXCR2 treatment was specific for neutrophils as T cell migration is not impacted following administration of this antibody [39].

We have shown that the T cell chemoattractant chemokine interferon-inducible protein 10 kDa (IP-10)/CXCL10 is rapidly expressed in response to JHMV infection and is strictly colocalized with viral RNA within the CNS [31]. Among the cell types responsible for CXCL10 expression, astrocytes were the primary cellular source as demonstrated through both in vitro and in vivo experiments [31]. Although not defined, we believe that expression of CXCL10 is in response to early expression of IFN-I as this cytokine has previously been shown to induce CXCL10 expression [40]. In addition to CXCL10, another T cell chemoattractant chemokine, CXCL9, is also expressed early in response to JHMV infection of the CNS [41]. Both CXCL9 and CXCL10 function by binding and signaling through the chemokine receptor CXCR3 which is expressed upon the surface of natural killer (NK) cells, activated CD4+ and CD8+ T cells, and antibody secreting cells (ASCs). We previously employed a recombinant strain of mouse hepatitis virus A59 (MHV-A59) that expressed CXCL10 from gene 4 of the viral genome to evaluate how CXCL10 shapes the innate immune response [42]. In brief, infection of the CNS of RAG1-/- mice (lacking functional T and B lymphocytes) with the CXCL10-expressing recombinant virus resulted in protection from disease associated with increased infiltration of NK cells into the CNS. Protection was mediated, in part, by secretion of IFN-*γ* which contributes to controlling viral replication within the CNS. However, the key functional role for both CXCL9 and CXCL10 is to attract virus-specific T cells (both CD4+ and CD8+ subsets) into the CNS by signaling through CXCR3. Through use of neutralizing antibodies specific for either CXCL9, CXCL10, or CXCR3 or employment of mice deficient in CXCL10, we demonstrated increased mortality accompanied by impaired ability to control replication within the CNS if these signaling pathways are disrupted [41,43,44,45]. The function of CXCL10 appears limited to the recruitment of activated T cells as antiviral effector functions e.g., proliferation, secretion of IFN-*γ*, and cytolytic activity remained intact [45]. In addition to recruiting activated T cells into the CNS, CXCL10 performs a similar function to attract ASCs into the perivascular space as well as parenchyma and this also aids in host defense by controlling viral replication [46]. Upon entry into the CNS, virus-specific T cells combat JHMV spread within the CNS through either secretion of IFN-*γ* or cytolytic activity [47,48]. CD4+ T cells are critical in not only directly controlling JHMV replication via secreting IFN-*γ* but also through providing support to CD8+ T cells. Studies by Hwang et al. [49] revealed that depletion of CD4+ T cells prior to infection did not significantly impact either the peripheral expansion, IFN-*γ* secretion, or recruitment of virus-specific CD8+ T cells into the CNS. Nonetheless, findings derived from this work revealed that CD4+ T cells are essential to prolong primary CD8+ T-cell function in the CNS as well as influencing memory CD8+ T cells for recall responses.

### 2.3. Microglial Involvement in JHMV Disease Progression

Resident glial cells clearly aid in host defense in response to microbial infection through various effector functions including secretion of IFN-I as well as proinflammatory cytokines/chemokines that assist in attracting cellular components of both the innate and adaptive immune response. However, more refined roles for resident glia in host defense in response to microbial infection are being discovered. As indicated above, production of IFN-I by activated microglia relies, in part, on prostaglandin D2 signaling via D-prostanoid receptor 1 (DP1) [38]. However, defining discrete functional roles of microglia in terms of host defense in response to JHMV infection is challenging in the face of infiltrating myeloid cells that may have overlapping functions. In the face of these challenges, Perlman and colleagues [50] provided an important study that further emphasizes that microglia are an active participant involved in an effective host response following JHMV infection of the CNS. Through use of an inhibitor of colony stimulating factor 1 receptor (CSF1R) that depletes microglia, yet only has a minimal effect on macrophages, it was determined that microglia play a critical role in both the early innate as well as virus-specific T cell responses. As early as day 4 postinfection, there is a dramatic increase in gene expression within microglia with the majority of genes being associated with IFN signaling and activation of IFN-regulatory factors. Depletion of microglia resulted in a dramatic increase in mortality that correlated with impaired ability to control viral replication within the CNS. In addition, microglia depletion led to increased infiltration of monocyte/macrophages into the CNS—although these cells had a less mature phenotype characterized by reduced MHC class II and elevated Ly6C expression. In addition, there was an overall increase in numbers of virus-specific CD8+ T cells yet there was an overall reduction in frequency and numbers of IFN-*γ*-producing CD4+ T cells and this likely contributed to the increase in viral titers within the CNS. Collectively, these findings illustrate important and previously unappreciated roles for microglia in contributing to protection from viral encephalomyelitis.

## 3. JHMV-Induced Demyelination

Although virus replication within the CNS is controlled by infiltrating virus-specific T cells, sterile immunity is not achieved and JHMV persists primarily in white matter tracts. JHMV persistence results in a chronic demyelinating disease in which loci of demyelination are associated with areas of viral RNA/antigen [51]. Clinically, mice develop loss of tail tone and a partial-to-complete hind-limb paralysis. Due to similarities in both clinical and histologic disease with the human demyelinating disease multiple sclerosis (MS), the JHMV model is considered a relevant animal model for studying mechanisms contributing to demyelination as well as remyelination [52,53,54]. A recent report detailed the genes and pathways associated with JHMV-induced demyelinating disease in the spinal cord. Through use of high-throughput sequencing of the host transcriptome, Weiss and colleagues [55] demonstrated that demyelination is accompanied by numerous transcriptional changes indicative of immune infiltration as well as changes in the cytokine production. Furthermore, these findings also supported a Th1-driven response that is associated with JHMV persistence and demyelination. 

Virally-encoded genes, notably for the spike glycoprotein, are important in JHMV neurovirulence and demyelination [56,57]. However, JHMV-induced demyelination involves immunopathologic responses directed against viral antigens expressed in infected tissues [58,59,60,61,62]. Inflammatory T cells and macrophages are considered important contributors to white matter damage in JHMV-infected mice. The importance of the immune system in driving demyelination in JHMV-infected mice is further emphasized by the demonstration that infection of RAG1-/- mice does not result in demyelination even though viral replication in resident glial cells, including oligodendroglia, is unrestricted. Adoptive transfer of either splenocytes derived from JHMV-immunized mice or virus-specific T cells results in spinal cord demyelination, further emphasizing the importance of T cells in driving disease. Secretion of IFN-*γ* by infiltrating T cells contributes to demyelination in JHMV-infected mice presumably through activating resident glia as well as inflammatory macrophages. The importance of both T cells and macrophages in contributing to demyelination in JHMV-infected mice is further highlighted by experiments in which targeting chemokines e.g., CXCL10 or CCL5 that attract activated T cells and macrophages limits the severity of white matter damage [63,64].

## 4. Neutrophils and JHMV-Induced Demyelination

While T cells and macrophages clearly are critical in contributing to demyelination in JHMV-infected mice, emerging evidence supports a role for other cell types that participate in white matter damage. For example, microglia have been argued to be important in demyelination through secretion of proinflammatory cytokines/chemokines as well as directed phagocytizing of the myelin sheath [65,66]. Emerging studies demonstrate an important role for neutrophils in experimental models of demyelination [67,68,69,70] and other models of CNS injury [71,72,73,74,75]. Neutrophil involvement has also been implicated in a number of systemic autoimmune diseases including systemic lupus erythematosus, rheumatoid arthritis, and anti-neutrophil cytoplasm antibody-associated systemic vasculitis [76]. Notably, neutrophils are a hallmark pathological feature of neuromyelitis optica (NMO) which is triggered by autoantibodies directed against the water channel aquaporin 4 expressed on astrocytes [77]. NMO lesions show accumulation of neutrophils in human patients while animal studies modeling NMO have found reduced neuroinflammation and myelin loss following treatment with neutrophil protease inhibitors [78,79]. Increasing evidence supports a role for neutrophil and/or neutrophil-derived molecules in amplifying the severity of white matter damage in human demyelinating diseases including MS [80,81,82,83,84,85]. Neutrophils are transient phagocytes that function as part of the innate immune system to respond to sites of injury and infection. Among the first responders following microbial infection, neutrophils enter into the bloodstream and follow chemotactic gradients to sites of injury within the CNS. CXCR2 binds the ELR+ family of chemokines including CXCL1 and CXCL2. Inflammatory events stimulate the release of granulocyte colony-stimulating factor (G-CSF) which in turn upregulates CXCL1, CXCL2, and CXCR2 while downregulating the CXCL12–CXCR4 axis that serves to retain neutrophils within the bone marrow. Circulating neutrophils must first attach to the vasculature before gaining entry into the CNS. Endothelial cells increase expression of transmembrane proteins including adhesion molecules (ICAM1 and VCAM1) and the neutrophil chemoattractants CXCL1 and CXCL2. These molecules attract and anchor neutrophils to the vasculature and this contributes to increasing the permeability of the BBB. Upon entry into the CNS, neutrophils continue to migrate to sites of infection/injury by responding to specific chemokine signals. Neutrophils have a potent antimicrobial arsenal including the release of reactive oxygen and nitrogen species that are toxic to many microbial pathogens. However, secretion of these molecules can lead to bystander damage instigating injury to surrounding host tissue. Indeed, preclinical mouse models of demyelination e.g., EAE and toxin models have demonstrated that neutrophils increase the severity of neuropathology and demyelination [86,87,88]. A better understanding of how neutrophils influence clinical disease and demyelination in preclinical models of MS is necessary to determine if these cells are relevant therapeutic targets.

### 4.1. Neutrophils in MS Patients

In MS patients, neutrophils are normally not detected in MS lesions and this most likely reflects their transient nature. Nonetheless, numerous studies have correlated neutrophil-associated factors with clinical disease in MS patients [83,85,89,90,91]. Higher levels of the neutrophil chemoattractant CXCL8 have been detected in the cerebrospinal fluid (CSF) of MS patients compared to healthy individuals. Additionally, CXCL8 levels measured through ELISA of CSF were higher in MS patients during relapsing episodes [92]. G-CSF, an important regulator for neutrophil trafficking from the bone marrow, was found to be upregulated in acute MS lesions taken from autopsy tissue [91]. Circulating neutrophils also exhibit a more primed state in MS patients characterized by higher expression of TLR-2, enhanced degranulation and oxidative burst, along with reduced apoptosis [83]. Neutrophil protease activity is also increased in MS patients experiencing a relapse compared to patients in remission or healthy controls. Moreover, relapsing MS patients showed increased plasma levels of CXCL5 during lesion formation. Expression levels of CXCL1, CXCL5, and neutrophil elastase also correlated with measures of MS lesion burden [85]. Collectively, these findings argue that neutrophils may contribute to disease progression in MS patients. 

### 4.2. Neutrophil Involvement in Preclinical Models of Demyelination

Supporting this notion is the demonstration that neutrophils have been shown to modulate demyelination in various preclinical models of MS. Liu and colleagues [86] found using the cuprizone toxin model of demyelination that *Cxcr2-/-* mice were resistant to demyelination. Furthermore, neutrophils were both necessary and sufficient in contributing to demyelination arguing CNS infiltration increased neurotoxic and inflammatory mechanisms which exacerbated toxin-induced demyelination. Further evidence for a role for neutrophils in augmenting demyelination is provided by Segal and colleagues [93] who demonstrated that *Cxcr2-/-* mice are relatively resistant to EAE and this correlated with reduced infiltration of neutrophils into CNS; however, transfer of CXCR2+ neutrophils into *Cxcr2-/-* mice immunized with encephalitogenic myelin peptides resulted in increased clinical disease and demyelination supporting the notion that neutrophils contribute to disease in EAE. Stoolman et al. [88] have expanded on these findings to show that enriched expression of CXCL2 within the brainstem attracts neutrophils that substantially contribute to the pathogenesis of EAE. Similarly, mice in which neutrophils lack suppressor of cytokine signaling 3 (SOCS3) exhibit an increase in susceptibility to the atypical EAE and this correlates with preferential recruitment of neutrophils into the cerebellum and brainstem [94]. The site of neutrophil recruitment may be critical in terms of amplifying histopathology as neutrophil accumulation within the brain, and to a limited extent in the spinal cord, contribute to tissue injury [87]. Collectively, these findings indicate that neutrophils can affect the severity of clinical disease and neuroinflammation in EAE.

In addition to EAE, TMEV infection of the CNS results in a rapid mobilization of neutrophils and monocytes that are recruited to the CNS. These cells are detected in the hippocampus of infected mice which is coincident with pathology. Targeted depletion of neutrophils/monocytes resulted in hippocampal neuroprotection and improved cognitive function [72]. Although the signaling mechanisms by neutrophils infiltrate into the CNS of TMEV-infected mice are not defined, the neutrophil chemoattractant chemokines CXCL1 and CXCL2 are secreted by astrocytes in response to infection suggesting these chemokines may function in attracting neutrophils into the CNS [95,96].

**Figure 1:**
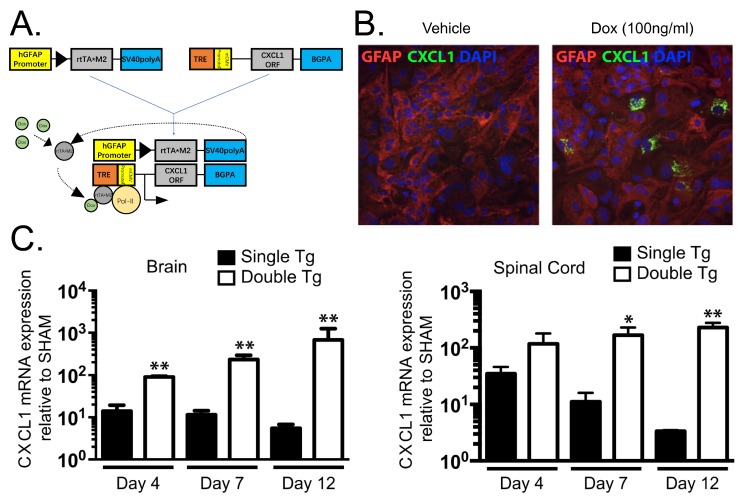
Derivation and characterization of a mouse model in which CXCL1 expression within the central nervous system (CNS) is under the control of a doxycycline promoter. (**A**) Cartoon depiction of experimental strategy to generate double (dbl) transgenic (tg) mice in which expression of mouse CXCL1 is under control of the glial fibrillary acidic protein (GFAP) promoter upon doxycycline treatment. (**B**) Cortex tissue from double tg and single tg postnatal day 1 (P1) mice was dissociated and enriched for astrocytes. Following 24-h of Dox (100 ng/mL) treated double tg astrocyte cultures, immunofluorescence confirmed CXCL1 expression within GFAP-positive astrocytes while vehicle treatment yielded no CXCL1 fluorescence (original magnification, ×20). (**C**) Within the spinal cord (SC) and brain, dox-treated double tg mice had statistically significant increases in CXCL1 mRNA expression over Dox-treated single tg mice at days 7 and 12 post-infection (p.i.) * *p* < 0.05, ** *p* < 0.01. Data derived from Marro et al., (2016) [101].

**Figure 2:**
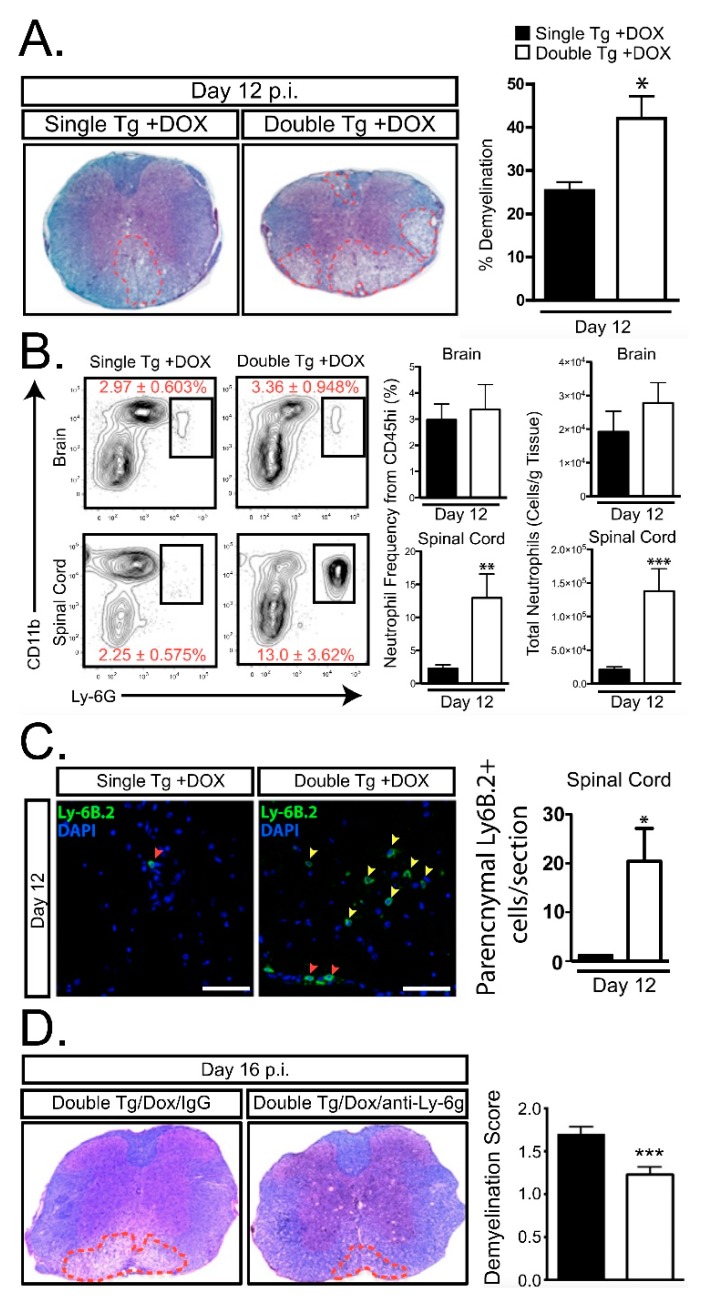
Elevated CNS CXCL1 expression is associated with increased neutrophil accumulation and demyelination. (**A**) Representative luxol fast blue (LFB)-stained spinal cords reveals increased (*p* < 0.05) demyelination in mouse hepatitis virus (JHMV)-infected Dox-treated double tg mice compared to single tg controls. **(B)** Flow cytometric analysis revealed a significant increase in the frequency and total number of neutrophils within the spinal cord of JHMV-infected Dox-treated double tg mice compared to single tg mice. (**C**) Representative immune-fluorescence staining further demonstrated a significant increase in the number of Ly6B.2-positive neutrophils (yellow arrowheads) within the spinal cord parenchyma of JHMV-infected double tg compared to single tg mice; red arrowheads indicate neutrophils located within the spinal cord meninges. Quantification of neutrophils within the spinal cords indicated an overall increase (*p* < 0.05) in Dox-treated double tg mice compared to Dox-treated single tg mice. (**D**) Representative LFB-stained spinal cord sections from JHMV-infected double tg mice treated with either control IgG2a or anti-Ly6G antibody between days 3 to 15 p.i. Quantification of the severity of demyelination revealed reduced white matter damage in mice treated with anti-Ly6G antibody compared to mice treated with isogenic IgG2a control antibody. * *p* < 0.05, ** *p* < 0.01, *** *p* < 0.001. Data derived from Marro et al., (2016) [101].

**Figure 3:**
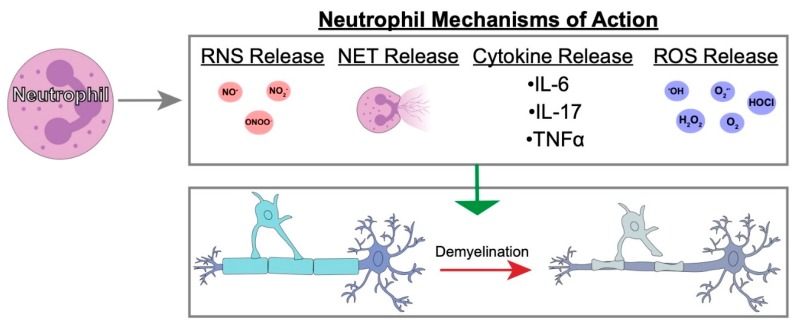
Characterization of possible neutrophil mechanisms of action that contribute to white matter damage. Cartoon depiction of neutrophil mechanisms of action including release of reactive nitrogen species (RNS) [105] and reactive oxygen species (ROS) [106] intermediates, neutrophil extracellular traps (NETs) [83], and select cytokines [107]. We hypothesize that these mechanisms may be responsible for the enhanced white matter damage observed following induced infiltration of neutrophils in preclinical mouse models of MS.

JHMV infection of the CNS results in the secretion of the ELR+ chemokines CXCL1, CXCL2, and CXCL5 at early times postinfection with virus [39,97]. Although we and others have determined that astrocytes express CXCL1 [31,97,98,99,100], it does not exclude the possibility that other resident CNS cells as microglia, neurons, and inflammatory immune cells are also capable of expressing CXCL1 as well as CXCL2 and CXCL5. These chemokines bind and signal through CXCR2 pathway to rapidly recruit neutrophils to the BBB. This also contributes to host defense by increasing BBB permeability via release of MMPs which subsequently enhances infiltration of virus-specific T cells [39]. Although neutrophils have been shown to contribute to clinical disease and white matter damage in EAE as well as toxin models of demyelination [86,87,88,93,94], the function of these cells in models of viral-induced demyelination have not been as well-characterized. To address this issue, we recently generated transgenic mice where targeted expression of CXCL1 in astrocytes is induced upon treatment with doxycycline (Dox) (Figure 1A,B) [101]. Treatment of JHMV-infected CXCL1 transgenic mice with Dox resulted in increased expression of CXCL1 mRNA transcripts and protein within the brain and spinal cords when compared to Dox-treated control mice (Figure 1C) [101]. Surprisingly, Dox-induced overexpression of CXCL1 within the CNS of transgenic mice did not influence expression of other proinflammatory cytokines/chemokines nor were there differences in inflammatory T cells into the CNS and control of viral replication within the CNS was not affected. Rather, there was a selective increase in neutrophil accumulation in the CNS and this was associated with an increase in clinical disease and demyelination (Figure 2A–C). Blocking neutrophil accumulation within the CNS of Dox-treated CXCL1 mice resulted in a significant reduction in demyelination further supporting a role for neutrophils in contributing to white matter disease (Figure 2D). Moreover, our lab has recently demonstrated similar results using this transgenic mouse model in the EAE model of demyelination. We observed that induced expression of CXCL1 in CXCL1-dg mice correlated with increased disease severity associated with neutrophil infiltration into the CNS and enhanced white matter damage. Blocking of neutrophil infiltration into the CNS ameliorated the severity of demyelination [102]. Importantly, we are now seeking to define the mechanisms by which CNS infiltrating neutrophils participate in white damage with a particular focus on secretion of reactive oxygen/nitrogen intermediates, neutrophil extracellular traps (NETs) as well as potentially activating resident glial cells and/or inflammatory monocytes/ macrophages [103,104] (Figure 3).

### 4.3. Possible Neutrophil Mechanisms of Action in Demyelination

Reactive oxygen and nitrogen species have been shown to be toxic to oligodendroglia and suggested to be involved in the pathogenesis of demyelination [108,109]. An additional neutrophil killing mechanism is the release of neutrophil extracellular traps (NETs). NET release is characterized by the neutrophil releasing DNA structures to ensnare foreign pathogens through chromatin decondensation. NETs can occupy 3–5 times the space as condensed chromatin. Different models of NETosis have been previously described including suicidal NETosis which occurs in a 2–4-h timeframe and vital NETosis in which nuclear or mitochondrial DNA is released within minutes to an hour following activation [110,111,112]. DNA is intrinsically toxic to microbes disrupting their membranes. Additionally, numerous neutrophil proteins also adhere to the expunged DNA including elastase and myeloperoxidase, which have their own antimicrobial effects. Viruses specifically have been investigated in relation to NET formation. Influenza, dengue, and human immunodeficiency virus 1 have all been shown to stimulate NET formation from circulating neutrophils. NETs have also been linked to a number of autoimmune diseases including psoriasis, rheumatoid arthritis, and systemic lupus erythematosus. However, little is known about NETs in relation to models of MS. Patient studies have shown higher circulating levels of NETs in serum from relapsing remitting MS patients compared to healthy controls, which has been suggested to aggravate tissue injuries [83]. Interestingly, a follow-up study by Sospedra and colleagues [113] found higher circulating NETs in male relapsing remitting MS patients compared to women suggesting an underlying sex-specific difference in pathogeneses. While current MS therapies focus on limiting infiltration of activated T cells into the CNS, the heterogeneous cellular nature of MS lesions argues that other cell types may be contributing to disease. As indicated above, neutrophils have been suggested to potentially participate in disease progression MS patients arguing that focusing on these cells may offer new therapeutic options for managing disease. Targeting neutrophil infiltration into the CNS through, for example, specific small molecule inhibitors that block chemokine receptor e.g., CXCR2 function may provide additional benefits when combined with existing disease modifying agents that limit the infiltration of circulating leukocytes. However, muting neutrophil recruitment to the CNS may have disadvantages as these cells are important in host defense against different neurotropic viruses and this approach may impact effective host responses.

## 5. Perspectives

The response of the innate immune system to viral-induced demyelination has been appreciated for a number of years, however new questions have arisen as to how neutrophils contribute to demyelination. Although the observance of neutrophils in MS patients has been elusive, likely due to their transient nature, patient samples indicate substantial evidence of neutrophil attractants and markers during disease. This is supported through evidence from several animal models of demyelination from our lab and others that have shown neutrophil recruitment into the CNS enhances demyelination. While the exact mechanisms of neutrophil contribution to demyelination remain obscure, recent studies employing autoimmune models of neuroinflammation/demyelination argue for a role for NETs and other neutrophil host defenses as possible instigators of damage. This information has emphasized the potential for targeting these cells as a therapeutic strategy to limit white matter damage.
